# Health Status in the Islamic Republic of Iran, Middle East and North Africa Countries: Implications for Global Health

**Published:** 2020-01

**Authors:** Hossein MIRZAEI, Zhaleh ABDI, Elham AHMADNEZHAD, Mahshad GOHRIMEHR, Elham ABDALMALEKI, Rezvaneh ALVANDI, Iraj HARIRCHI

**Affiliations:** 1 HIV/STI Surveillance Research Center, WHO Collaborating Center for HIV Surveillance, Institute for Futures Studies in Health, Kerman University of Medical Sciences, Kerman, Iran; 2 National Institute for Health Research (NIHR), Tehran University of Medical Sciences, Tehran, Iran; 3 School of Medicine, Tehran University of Medical Sciences, Tehran, Iran

**Keywords:** Health status, Non-communicable disease, Iran, Global health, Sustainable development goals

## Abstract

**Background:**

The aim of this study was to evaluate the health status of the Iranians following the sustainable development goals’ (SDGs) introduction and the recent health reform implementation in Iran and to compare with those of the Middle East and North Africa region (MENA) and global average.

**Methods:**

This comparative study used secondary data to investigate socio-demographic and health status indicators. The sources included census, population-based surveys and death registries. Global and regional health status indicators were obtained from international databases including WHO, the World Bank and the Institute for Health Metric and Evaluation (IHME).

**Results:**

Life expectancy and human development index improved following the reform implementation. Among causes of death, 74.6% were attributed to non-communicable diseases (NCDs). There was an increasing trend in risk factors for NCDs in Iran, while at the same time neonatal, infant and under-5 mortality rates reduced. Compared to the MENA, Iran has a lower maternal mortality ratio, neonatal, infant, and under-5 mortality rates, and a higher life expectancy. NCDs and road traffic injuries accounted for a larger portion of disability-adjusted life years in Iran compared to the MENA and worldwide.

**Conclusion:**

Actions against communicable diseases and road traffic injuries are required together with continued efforts to address NCDs. Although based on the results, Iran has relatively high rankings, there is a need to develop a roadmap to accelerate achieving global health goals and SDGs targets.

## Introduction

The burden of infectious diseases has reduced globally through the strengthening of the health systems and development of primary health care (PHC) strategy (1, 2). However, health systems still face a number of challenges, which include demographic and epidemiological transitions and shifting disease burden from communicable to noncommunicable diseases (3). SDGs, introduced as a main post-2015 agenda, will enable countries to provide health for all (4). Health is placed centrally within the SDGs (5, 6).

The Alma-Ata Declaration was the first international declaration that advocated PHC (7). Improvements in health status following PHC implementation in Iran have changed disease patterns (8). Forty years after the launch of PHC, universal health coverage (UHC) as the central target of SGDs, has been given a prominent place in the post-2015 global agenda. Countries have gradually put the program on their agenda since 2000 (9). As many other countries, Iran is committed to achieve UHC. Therefore, a health reform, called Health Transformation Plan (HTP), was initiated in early 2014 with the prime intention to achieve UHC. With a considerable increase in the Ministry of Health and Medical Education (MoHME)’s budget, insurance coverage was extended to approximately 10 million people. A number of other interventions were introduced to reduce Iranian out of pocket (OOP) payments to 20% or less of the Total Health Expenditure (THE). A few months later, the next phase of the HTP started scaling up the primary health services coverage. One of the main interventions implemented in Oct 2014 to reduce OOP payments was updating relative value units of health services to better reflect the cost of services in both public and private sectors (6). Health status indicators are employed as benchmark to track progress towards achieving broad health system objectives as well as evaluate the performance of health systems and the success of health programs. Health indicators are also employed to examine the differences in health status within and between countries (9).

In this study, health status indicators were used as benchmark to determine whether the stated goals of health programs and UHC in Iran have been achieved. Furthermore, the indicators of Iran were compared with those of countries located in the MENA and the global average.

## Methods

This comparative study used secondary data analysis. The indicators definitions and data collection methods are as follows:

### Socioeconomic indicators

1. **Demographic indicators**
  The components are: i) Total population, ii) Average population growth rate, iii) Population over 65 yr of age, iv) Urban population in Iran in 2016 were compared with those in the MENA and the world. Data for MENA and global average was obtained from the World Bank database and for Iran collected from censuses conducted in 2006, 2011 and 2016.
2. **Human Development Index (HDI)**
  HDI is a summary measure of long and healthy life, being knowledgeable and have a decent standard of living. The health dimension is assessed by life expectancy at birth; the education dimension is measured by mean of years of schooling for adults aged 25 yr and more and expected years of schooling for children of school entering age. The standard of living dimension is measured by gross national income per capita (GNI) (based on 2011 Purchasing Power Parity) (1, 5, 10). Data on HDI were collected from the United Nations database in 2015.

### Health status and risk factors

**1. Life expectancy (LE)**
  - **LE at birth**
    It is the average number of years that a newborn could expect to live if he or she were to pass through life exposed to the sex- and age-specific death rates prevailing at the time of birth, for a specific year, in a given country, territory or geographical area. LE at birth is derived from life tables and is based on sex- and age-specific death rates. United Nations (UNs) values for LE at birth correspond to mid-year estimates, consistent with the corresponding UNs fertility medium-variant quinquennial population projections. The IHME database was used to compare LE in Iran to those of MENA and global average in 2015 (5,10).
**2. Main causes of death and disease burden**
  - **Main causes of deaths**
    Measuring how many people die each year and why they have died, is one of the most important means –along with gauging how various diseases and injuries are affecting the living– for assessing the effectiveness of a country’s health system. The indicators for Iran were obtained from the World Health Organization (WHO) (10). The indicators for the MENA and the world were extracted from IHME database.
  - **Years of healthy life lost to premature death and disability (DALYs**)
    DALYs are the sum of years of life lost (YLLs) and years lived with disability (YLDs) (1, 10). Data sources were the same as those for main causes of deaths.
**3. Neonatal, infant and under-5 mortality rates**
  - **Neonatal Mortality Rate (NMR)**
    It is the number of deaths during the first 28 completed days of life per 1000 live births in a given year or period. The number of children who died during the first 28 days of life should be divided by the number of live births in 1000 births (years of exposure) (1). Data from Iran's Multiple-Indicator Demographic and Health Survey (IrMIDHS conducted in 2000IrMIDHS conducted in 2010), surveillance routine systems and death registries were used to capture the indicator information for Iran. The World Bank database was used to check these indicators in the MENA and the world.
  - **Infant Mortality Rate (IMR) and Under-5 mortality rates (U-5 MR)**
    It is the probability that a child born in a specific year or period will die before reaching the age of 1 (for IMR) and 5 (for U-5MR) year, if subject to age-specific mortality rates of that period, which is expressed as a rate per 1000 live births. To calculate, the number of children who died before their first birthday (0–11 months of age) should be divided by the number of live births (years of exposure) (1). For U-5MR, the number of deaths among children aged 0–4 yr (0–59 months of age should be divided by the number of live births (person-years of exposure). Data sources were the same as those for NMR.
4. **Maternal Mortality Ratio (MMR)**
  It is the number of maternal mortality per 100,000 live births during a specified time, usually one year. The number of maternal deaths should be divided by the number of live births used to calculate the indicator (5). The WHO mortality database was the main source for MMR.
**5. Risk Factors**
  - **Tobacco use**
    The “prevalence of current tobacco use” was estimated using two main indicators. Current daily tobacco use for all ages in 2016 extracted from IHME database. Trends of current daily and non-daily tobacco use (18+ yr) were extracted from NCDs Risk Factor Surveys (STEPs) conducted in 2005, 2010 and 2016 in Iran.
    **Prevalence of overweight in persons aged 18+ yr**
    It is the percentage of adults (18+ yr) who are overweight (having a BMI ≥ 25 kg/m^2^) and obese (having a BMI≥ 30 kg/m^2^) (3, 5). WHO database was used to estimate the prevalence of overweight for Iran, MENA and global average in 2016.
    To investigate the trends of overweight and obesity (18+ yr) in Iran, data from STEPs conducted in 2005, 2010 and 2016 were used.
    **Raised blood pressure (BP) and Blood glucose**
    Prevalence of raised BP among persons aged 18+ yr is defined as systolic BP≥140mmHg and/or diastolic BP≥90mmHg. Number of respondents with systolic BP≥140mmHg or diastolic BP≥90mmHg was divided by all respondents of the survey aged 18+ yr used to calculate the indicator. A fasting blood sugar level higher than 126 mg/dl considered as diabetes (3, 5). Data sources were the same as those for overweight.

All estimations were adjusted based on the population size. There was no possibility for age and sex adjustment due to data non-availability.

The following 19 countries of the MENA were included in this study: Algeria, Bahrain, Djibouti, Egypt, Iran, Palestinian territories, Iraq, Jordan, Kuwait, Lebanon, Libya, Morocco, Oman, Qatar, Saudi Arabia, Syria, Tunisia, the United Arab Emirates and Yemen.

### Ethical approval

Ethics approval for this study was obtained from Research Ethics Committee of the National Institute for Health Research (Ethics ID: IR.TUMS.NIHR.REC.1396.36).

## Results

### Socioeconomic characteristic

**Demographic indicators**: Iran, accounts for 18.3% of MENA and 1% of the world's population. The annual population growth rate (GR) in Iran is 1.24%, which is lower than that of the MENA (1.73%), and is higher than the annual population GR in the world (1.15). The proportion of the population aged 65 yr and above in Iran is 6.1%, which is higher than the MENA average and lower than the global average. In 2016, the percentage of the total population living in urban areas of Iran was 74%, which is higher than the average urbanization rate in the MENA and the world (Table 1).

**Table 1:** Demographic and Socioeconomic indicators in Iran, MENA and the world

***Variable***	***IRAN and Rank (In MENA)***	***MENA***	***Global***
Population (2016) – (×1000)	79,926 (2)	436,720	7,442,135
65yr and above (% of total) (2016)	6.1 (6)	4.85	8.48
Urban population (% of total) (2016)	74 (14)	64.5	54.29
HDI (2015)	0.774 (8)	0.709	0.717
Mean years of schooling (2015)	8.8 (8)	7.3	8.3
GNI per capita (2016)	16,395 (8)	17,308	14,447

**Human development:** The HDI in Iran is 0.774 (2015), which is higher than the global average and MENA’s average. Iran ranks 8^th^ in the MENA and 69^th^ in the world in terms of HDI. The average years of schooling in Iran is 8.8 yr.

GNI per capita based on purchasing power parity (PPP- constant price of the international dollar in 2011) is 16,395 in Iran, which is higher than the world's GNI per capita and is lower than the average in the MENA. According to the table (trends of indicators), Iran has an increasing population GR with a simultaneous growing of elderly population (Table 2).

**Table 2:** Trends in Demographic and Socioeconomic indicators in Iran

***Indicator***	***2000***	***2010***	***2015***
Population– (×1000)	70,495	75,149	79,926
65y and above (% of total)	5.2	5.7	6.1
Mean age of population	24.7	29.8	31.1
Urban population (% of total)	68.9	71.4	74
HDI -2015	0.666	0.755	0.774
Mean years of schooling	6.2	8.2	8.8
GNI per capita	11,348	17,520	16,395

### LE, Child and maternal mortality

**Fig. 1:**
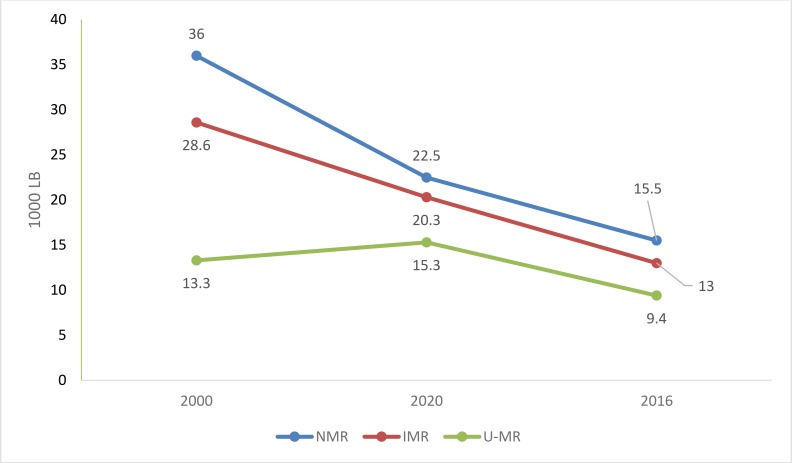
Trends in neonatal, infant and under 5 mortality rates in Iran

In 2016, U-5MR in Iran was 15.5 per 1000 live births. The IMR was 13 per 1000 live births. The NMR was 9.6 per 1000 live births. The U-5MR and NMR decreased in 2016 compared to 2010 and 2005 in Iran (Fig. 1).

In 2016, the MMR in Iran was 19 per 100,000 live births, which is lower than the average MMR in the MENA (81) and the world (216). The MMR decreased by 33% from 2010 (28.4) to 2016 (19). It decreased by 60% from 2000 (48) to 2016 (19). LE at birth in Iran is 75.6 yr in 2016, and was 71.4 yr in 2000 and 73.7 yr in 2010 (Table 3).

**Table 3:** LE, child mortality rates and MMR Iran, MENA and the world

***Variable***	***IRAN and Rank (In MENA)***	***MENA***	***Global***
LE -2016	75.5 (8)	73.2	72.5
HALE at birth -2015	64.79 (10)	62.5	63.12
NMR -2016	9.6 (12)	14.6	18.6
IMR -2016	13 (12)	20.1	30.5
U-5MR -2016	15.5 (12)	24	40.8
MMR -2016	19 (10)	81	216

### Mortality and burden of disease

**Fig. 2:**
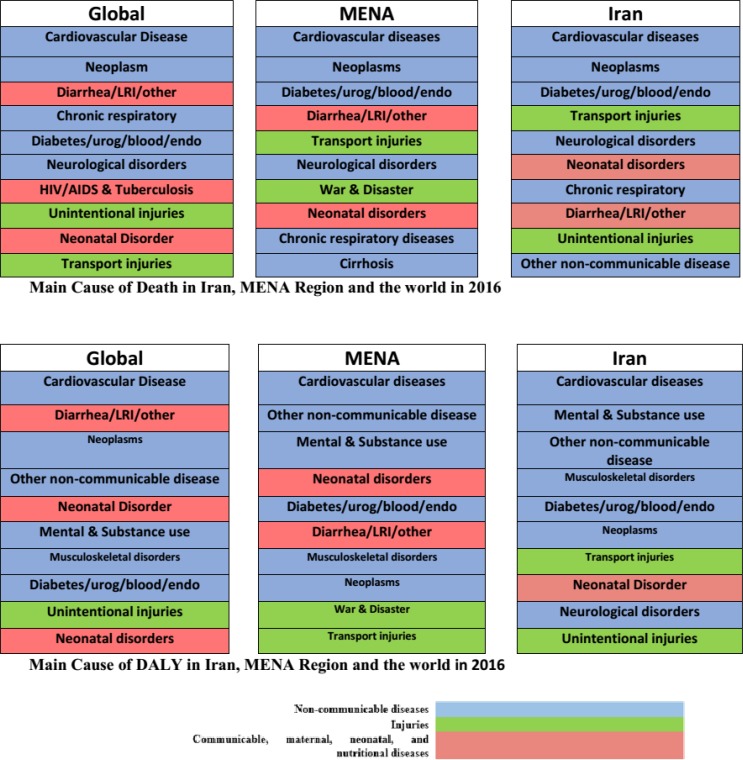
Main causes of DALY in Iran, MENA and the world in 2016

In 2016, 79.7% of all deaths and 74.1% of DALYs were due to NCDs. In the MENA, these figures were 72.2% and 63.9%, respectively. Globally, 72.2% of all deaths and 61.3% of DALYs were attributed to NCDs (Fig. 2).

Infectious diseases, maternal and child mortality; and nutritional factors account for 7.7%, 13.2% and 19.3% of deaths in Iran, MENA and the world, respectively (Fig. 2).

### Risk factors

**Fig. 3:**
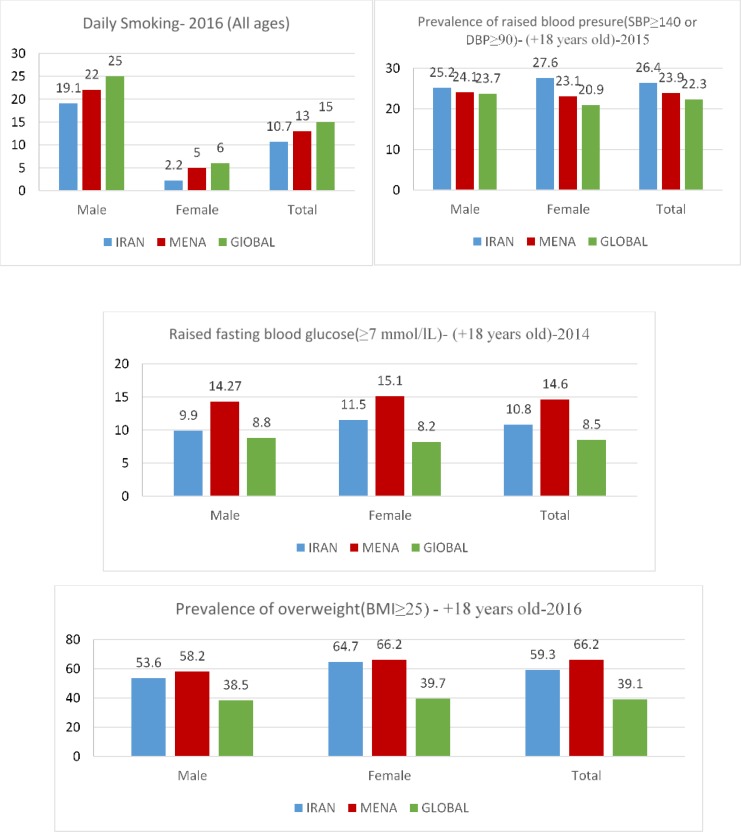
Prevalence of NCDs risk factors in Iran, MENA and the world (last available data)

**Fig. 4:**
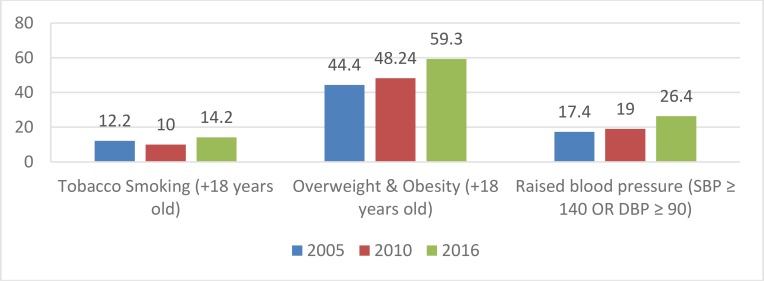
Trend of risk factors for NCDs in Iran

Figure 3 shows the prevalence of NCDs risk factors. Figure 4 compares the prevalence of smoking, overweight and obesity and raised BP in 2005, 2010 and 2016 in Iran. The prevalence of all NCDs risk factors increased in 2016 compared to 2005. The prevalence of obesity and overweight (BMI> 25) among Iranians over the age of 18 yr was 59.3%. The prevalence of overweight and obesity increased in 2016 compared to 2005. The prevalence of hypertension among males and females was 25.2% and 27.6%, respectively. The prevalence of high blood sugar in males and females was 9.9% and 11.5%, respectively.

## Discussion

The aim of the study was to compare Iran’s health indicators with the health indicators of countries located in the MENA and the global average. Compared to the MENA, Iran has a lower maternal mortality ratio, neonatal, infant, and U-5MR, and a higher LE and HDI. LE and HDI improved following the HTP implementation. Among causes of death, 74.6% were attributed to NCDs. There was an increasing trend in risk factors for NCDs in Iran, while at the same time NMR, IMR and U-5MR reduced. NCDs and road injuries accounted for a larger portion of DALYs in Iran compared to the MENA and worldwide.

Measurements of health status allow the identification of health priority issues as well as monitor the progress made towards achieving the SDGs and UHC (5, 6). Determination of health status indicators can help to set benchmark for attaining UHC. It can also assist with monitoring of health status trends and providing benchmarks for assessing the success of programs in achieving the desired targets (9, 10).

The indicators’ trends suggested that LE increased following the implementation of the HTP. There was also an improvement in the mortality indicators during this period. However, there was an increase in the contribution of NCDs and their underlying risk factors to the burden of disease during this period. Given the relatively short period between the HTP implementation and evaluation, these changes may not be attributable to the introduction of the HTP. Nevertheless, this study can be used as a benchmark to evaluate the long-term effects of future reforms.

Health status indicators of Iran were compared with the average of the MENA and global. This helps to compare Iran’s health status indicators with the region’s average at a glance. In addition to the region’s average, we compared Iran's health status indicators with those of every single country located in MENA region. We did this for two reasons. The first reason is related to the mean property, heavily influenced by outliers. If the situation of one or more countries is much different from other countries, it can influence the average. Secondly, countries located in the MENA are significantly different in terms of socioeconomic status and health status. For instance, there is a wide variation across countries’ HDI such that there are countries with very high HDI or very low HDI in the region. Therefore, Iran’s indicators should be compared with each country’s indicators.

Maternal, infant, neonatal and U-5MR in Iran are lower than the MENA and global average. LE at birth was higher than the MENA and global average. The trend of maternal and child mortality in Iran is on the decrease, consistent with previous studies (11, 12). This significant improvement in the health status indicators can be attributed to the successful implementation of the PHC program in Iran (13). In terms of the HDI, Iran occupies the eighth position in the region.

Palestinian territories, Qatar, Saudi Arabia, UAE, Bahrain, Kuwait and Oman have HDI higher than 0.8 and are among the countries with very high HDI. Iran, Algeria, Lebanon, Jordan, Tunisia and Libya have a HDI between 0.7 and 0.799 and are considered to have a high HDI. Egypt, Iraq and Morocco, which have HDI between 0.55 and 0.69, are among the countries with medium HDI. In Syria, Sudan, Yemen and Djibouti, HDI is below 0.55. These countries are considered to have low HDI. There was a change in HDI ranking in the MENA in 2015. Saudi Arabia witnessed the highest increase in HDI value during 2010–2015 and ranked 38^th^ in the 2015 world rankings. Moroccan HDI ranking improved four places, Iran HDI ranking improved three places, and Qatar HDI ranking improved two places during that period. In contrast, the HDI ranking of countries such as Libya, Syria, Yemen and Lebanon dropped by 35, 29, 12 and 12 points, respectively (14).

The impact of war on health and consequently on HDI must be taken into account while considering countries HDI (15, 16). Review of changes in HDI index shows that although the ranking of most countries in the MENA changed minimally, HDI in countries like Libya, Syria, Lebanon and Yemen changed tremendously (12 to 35 ranks). A review of the causes of death in these countries showed that although in the majority of MENA countries, cardiovascular diseases are the main causes of mortality and morbidity, civil war and conflict in Syria was the first cause of death (45% deaths); in Iraq and Yemen, it is the second leading cause of death and in Libya, it is the fourth cause of death (10). Iran’s HDI has increased favorably and, it is anticipated that by 2030 (targeted year to achieve SDGs) Iran will be among the countries with a high HDI.

NCDs and road accidents in Iran account for more deaths and Years of Life Lost (YLL) due disability compared to the MENA and the world. This could be due to the low mortality induced by infectious diseases, maternal mortality, child mortality and nutritional factors. The results of the current study and those of other studies (17) showed that the burden of NCDs continue to rise rapidly in Iran and the MENA. The number of premature deaths due to NCDS will increase significantly in the future. Furthermore, there is a difference in the ranking of the causes of death in Iran, the MENA and the world at large. Deaths from disasters and war are not among the 10 leading causes of death and disability in Iran and the world at large. However, in the MENA, it is the 7^th^ leading cause of death and disability. This is due to civil wars and conflicts in four MENA countries: Syria, Iraq, Yemen and Libya. Syria’s civil war accounted for 45% of deaths and 47.2% of DALYs in 2016. It accounts for 11.1% of deaths and 12.3% of DALYs in Iraq, 14.4% of deaths and 13.3% of DALYs in Yemen. However, in Iran, war and disasters accounts for 0.18% of deaths and is the 20^th^ leading cause of death. It accounts for 0.65% of the DALYs and twenty-first leading cause of DALYs (10).

Given the growing burden of NCDs, in 2013, WHO initiated a global action plan to prevent and control NCDs. The most important objective is a 25% reduction in the deaths from NCDs (13). Following the introduction of HTP, national action plan was developed to monitor and assess all measures aimed at controlling NCDs and risk factors in Iran (18). The plan was aimed at rejigging health systems to include NCDs and mental health services in PHC services. Although many proven interventions for NCDs were considered in the plan, more targeted interventions are needed to reduce the risk factors of NCDs among all subgroups of population. The effects of these interventions should continuously be monitored and evaluated.

This study had some limitations that future studies can take into account. We studied only the health status indicators and did not include health systems financing indicators. For future studies, we recommend to include Iran’s health system financing indicators and compare them with those of MENA countries. The impact assessment of the HTP was not carried out in the current study due to the aim of paper. Hence, the HTP effects on health indicators and its weaknesses or strengths compared to other health programs conducted in the other countries will can be investigated more in future.

## Conclusion

Indicators of child and maternal mortality in Iran are lower than MENA and global average. Indicators related to HDI in Iran was better than MENA and global average. Main causes of death are approximately the same in Iran, MENA and worldwide, but there are some differences such as war and disaster that is the seventh cause of death in MENA, but is not between the 10 top causes of death in Iran and in the world. Death from HIV/AIDS in Iran and MENA is not among the first 10 causes of death, but globally it is the seventh cause of death. Tobacco use in Iran was lower than the global and the MENA average and the prevalence of obesity and overweight (BMI> 25) among Iranians was lower than the MENA and higher than the global average. The prevalence of hypertension among Iranian is higher than that of the MENA and the global average. International organizations should play a constructive role in the support of peace in the region and enhance their role in the fight against terrorism, which can be very effective in improving health and reducing mortality among the involved countries.

## Ethical considerations

Ethical issues (Including plagiarism, misconduct, data fabrication and/or falsification, double publication and/or submission, redundancy, etc.) have been completely observed by the authors.
